# Toxicity Assessment of Expired Pesticides to Green Algae *Pseudokirchneriella subcapitata*

**DOI:** 10.5402/2012/247072

**Published:** 2012-11-14

**Authors:** G. Satyavani, G. Chandrasehar, K. Krishna Varma, A. Goparaju, S. Ayyappan, P. Neelakanta Reddy, P. Balakrishna Murthy

**Affiliations:** ^1^Toxicology, International Institute of Biotechnology and Toxicology (IIBAT), Padappai, Kancheepuram District, Tamil Nadu 601301, India; ^2^Bio-organic Chemistry, Central Leather Research Institute, Adayar, Chennai, Tamil Nadu 600 020, India

## Abstract

In order to investigate the effect of expired pesticides on the yield and growth rate of green algae *Pseudokirchneriella subcapitata*, a study was conducted as per the Organisation for Economic Cooperation and Development (OECD) guideline number 201. Fifteen expired pesticide formulations, most commonly used in Indian agriculture, were tested in comparison with their unexpired counterparts. The expired pesticide formulations studied belonged to various class and functional groups: organophosphate, pyrethroid-based insecticides; azole-based fungicides; acetamide, propionate, acetic acid-based herbicides; fungicides mixtures containing two actives—azole and dithiocarbamate. The toxicity endpoints of yield (E_y_C_50_: 0–72 h) and growth rate (E_r_C_50_: 0–72 h) of *Pseudokirchneriella subcapitata* for each pesticide formulation (both expired and unexpired pesticides) were determined statistically using TOXSTAT 3.5 version software. The results pointed out that some expired pesticide formulations exhibited higher toxicity to tested algal species, as compared to the corresponding unexpired pesticides. These data thus stress the need for greater care to dispose expired pesticides to water bodies, to avoid the effects on aquatic ecospecies tested.

## 1. Introduction

Agriculture is the back bone of world economy, and in India about 60% of the population depends on agriculture as their only occupation [1]. Pesticides have been a major contributor to the growth of agricultural productivity and food supply [2]. India ranks 10th position in the world in pesticide consumption as its total consumption amounts to about 500 million tons. India is presently the largest manufacturer of pesticides among the South Asian and African countries, with the exception of Japan. The Indian pesticides market is the 12th largest in the world with a value of US $0.6 bn, which is 1.6% of the global market pie [3]. Process of agricultural production is supported by the increased use of agrochemicals [4]. The increased use of pesticides will cause an increase in the number of pesticide dealers, including fumigators/pest controllers, retailers, and formulators [5]. Majority of dealers were reportedly interested in profit making and not pest control [6]. In India, obsolete pesticides have been either repacked or their date of expiry been erased and a new date of expiry given, and such pesticides are sold at a reduced price in the pesticide mela during festival season [7]. A recent case reported by Meenakam, World Tamils News Media, Hayleys Agro Chemical Company, Brahmanagama was caught while changing labels of expired pesticides from the date of expiry 2008 to 2012. On July 12, 2011, The Sri Lankan Consumer Affairs Authority raided the warehouse of the company and found 100,000 expired packets of pesticide, worth Rs. five million [8].

A case was reported at Nongpoh, Shillong, were packets of expired pesticides were distributed free of cost to farmers. According to the officer, the pesticide was supplied by M/s. ENBEE Enterprise of Shillong in the month of July 2010. However, the farmer received the expired pesticide in the month of March, 2011 [9]. Another case of selling expired pesticides illegally by a dealer at Punjab in 2002 was reported. The city police registered a case against Bhim Sain Goyal, a pesticide dealer and former president of the Mansa Fertilisers, Seeds and Pesticides Dealers Association for selling expired, substandard, and spurious pesticides. According to the First Information Report, the dealer had changed the date of manufacturing, expiry, and the price of pesticides. The pesticides had already expired on the date of sale to the farmer by the owner of Union Seed Agency [10]. Expired pesticides are the obsolete pesticides which are no longer used for original purpose and these obsolete pesticides are a major threat to human health and environment. There are stocks of pesticides that have lost their efficacy because their shelf life has expired. The expiry date indicates the endpoint when a chemical is no longer within acceptable specifications for its efficiency and stability [11]. The terms “expired” and “obsolete” pesticides may be used interchangeably as in practical terms the two situations are the same [12]. India has at least 200 tons of obsolete pesticides in terms of active ingredients [13]. In Nepal, the amount of expired pesticides was estimated as 150 tons; however, despite the date of expiry being reached, 39 tons were used in farming and 75 tons were sprayed without planning or buried [14]. 

 Due to lack of awareness, farmers are using these expired pesticides which will enter water bodies through agriculture runoff. A study conducted in Rift Valley, Ethiopia, showed that 62% of farmers did not check the expiry date of the pesticides when they bought them and 10% of the farmers kept left-over pesticides and used them in the next season [15], which were expired when used. PAN UK reported that about 91% of the farmers prepared their pesticides close to water sources used by local people for drinking, cooking, and other household purposes. In all, 61% washed their pesticides sprayers and other equipment on the farm field [15] in Ethiopia. EPA in 1990 reported that 10% of community wells and 4% of rural domestic wells have detectable levels of at least one pesticide of the 127 pesticides tested in a national survey [16]. Along with this, obsolete pesticides are more likely to be spilt or leak into the environment and populations and the risk of exposure to these pesticides is higher [17]. Leaking of disposed date expired pesticides, runoff of sprayed chemicals, and improper disposing of empty containers are some of the sources which pose major threat to water contamination [18].

 Algae are one of the essential living organisms of aquatic ecosystems. They produce oxygen and organic substances on which most other life forms depend by providing food for other organisms, including fish and invertebrates. Toxic chemical effects on algae can directly affect the structure and function of an ecosystem, resulting in oxygen depletion and decreased primary productivity [19], and also affect the food chain in the aquatic ecosystem. Natural ecosystems are complex and consist of many layers of interacting organisms. Damage to any one of these organisms in the ecosystem may have an impact on the entire ecosystem [20]. Pesticides can affect the structure and function of aquatic communities through altering species composition of an algal community [21]. Organophosphate pesticides and other pesticides (such as pyrethroid-based pesticides) used in urban and agricultural areas in the SJR (San Joaquin River) watershed and Delta are causing aquatic life toxicity in the state's waters [22]. Pesticide mixtures in streams draining agricultural watersheds resulted in increased risk compared with consideration of individual pesticides to *Pseudokirchneriella subcapitata* [23]. As a threat reported to green algae, we focused on the toxicity of expired pesticides towards green algae *Pseudokirchneriella subcapitata*.

## 2. Objective

 The purpose of the present study is to determine the toxicity of the expired and unexpired pesticide formulations towards *Pseudokirchneriella subcapitata* and compare them with their toxicological endpoints such as E_y_C_50_ and E_r_C_50_ and their 95% confidence limits. Growth is quantified with measurements of the algal biomass as a function of time.

## 3. Materials and Methods

 Based on the wide usage of pesticide formulations in India, the following pesticide formulations were selected for the present study: Insecticides: (1) Dichlorvos 76% EC, (2) Endosulfan 35% EC, (3) Quinalphos 25% EC; Pyrethroids: (4) Alphacypermethrin 10% SC, (5) Fenvalerate 20% EC, (6) Lambda-cyhalothrin 5% EC; Herbicides: (7) Pretilachlor 50% EC, (8) 2,4-D sodium salt 80% WP, (9) Fenaxaprop-p-ethyl 9.3% EC;Fungicides: (10) Tebuconazole 25% EC, (11) Mancozeb 75% WP, (12) Hexaconazole 5% EC; Combinational Fungicides: (13) Captan 70% + Hexaconazole 5% WP, (14) Carbendazim 12% + Mancozeb 63% WP, (15) Metalaxyl 8% + Mancozeb 64% WP. 

 All the above products (unexpired pesticide formulation) were purchased from pesticide shops in the market, and all the products were within the mandatory for two-year-shelf life period. The same 15 expired products were obtained from IIBAT's repository (expiry period within the range of 10 to 24 months after two years of shelf life) were tested simultaneously as given in Table 1. Manufacturer's name and brand name of pesticides are withheld.

 The test species green alga *Pseudokirchneriella subcapitata* strain SAG 61.81 was used in this study. The primary culture was procured from University of Göttingen, Germany, and the culture is maintained in the Department of Ecotoxicology, IIBAT, as per the procedure stated in the OECD guideline number 201 [24]. 

 The nutrient culture medium, OECD TG 201 medium (Table 2), was used for the present study. The deionised water was used to prepare OECD medium throughout the study. All pesticide formulations were dissolved in the OECD medium without using any solvent. The initial pH of bulk solution of each test item concentration and control was recorded and adjusted when required to 8.1 ± 0.1 using 0.1 M NaOH (sodium hydroxide) solution, and final pH was recorded in control and treated flasks at 72 h after incubation. The test item concentrations (Table 3) were fixed for each pesticide formulation both for expired and unexpired pesticide formulations on the basis of available reference literature for unexpired pesticide formulation [25]. The test item stock solution was diluted with the OECD medium to attain the required test item concentrations. For each treatment and control, 100 mL of test item solution was transferred into sterile 250 mL Erlenmeyer flasks. 

 The control and treatment flasks were inoculated with three-day-old preculture of *Pseudokirchneriella subcapitata* to get an initial cell concentration of about 1 × 10^4^ cells per mL under aseptic conditions. All the flasks were kept in the shaker incubator for 72 h providing continuous illumination of 6500–8000 lux light intensity with temperature ranging 20–22°C and continuously shaken at 110–120 rpm. During the experiment, control and treatment flasks were randomly repositioned in the shaker incubator daily to minimize the variation of the light intensity. After 72 h of incubation, alga cell count was recorded in all the flasks using Improved Neubaur's Haemocytometer under illuminance of the microscope. 

 Based on the inhibitions of yield and growth rate, E_y_C_50_ (0–72 h) and E_r_C_50_ (0–72 h) were determined with 95% confidence limits by probit, logit, and log-log model. The EC_X_ (effective concentration) values were reported based on the statistical model with the best fit. The calculations were done using software TOXSTAT 3.5 version [26].

## 4. Results and Discussion

**Figure 1:**
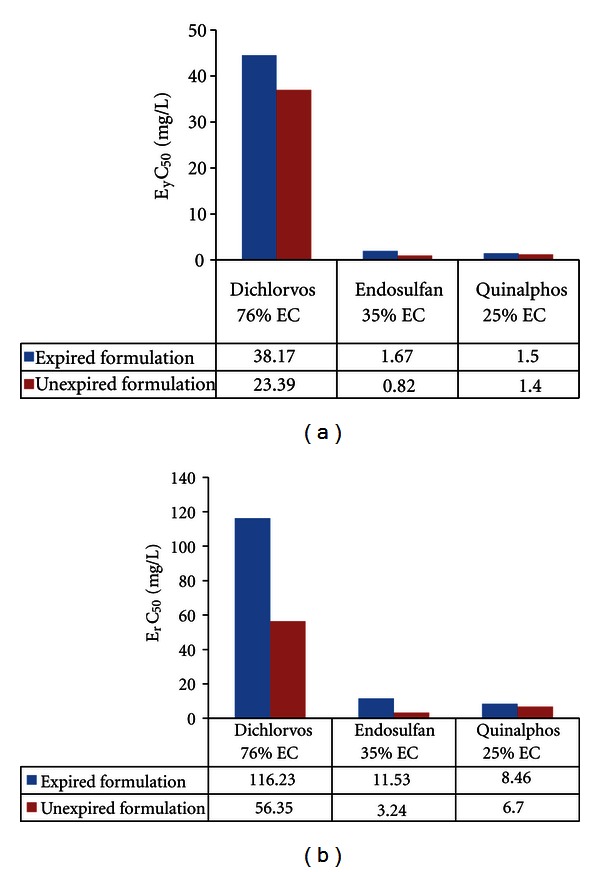
Effective yield and growth rate concentration of insecticides on *Pseudokirchneriella subcapitata* at 0–72 h.

**Figure 2:**
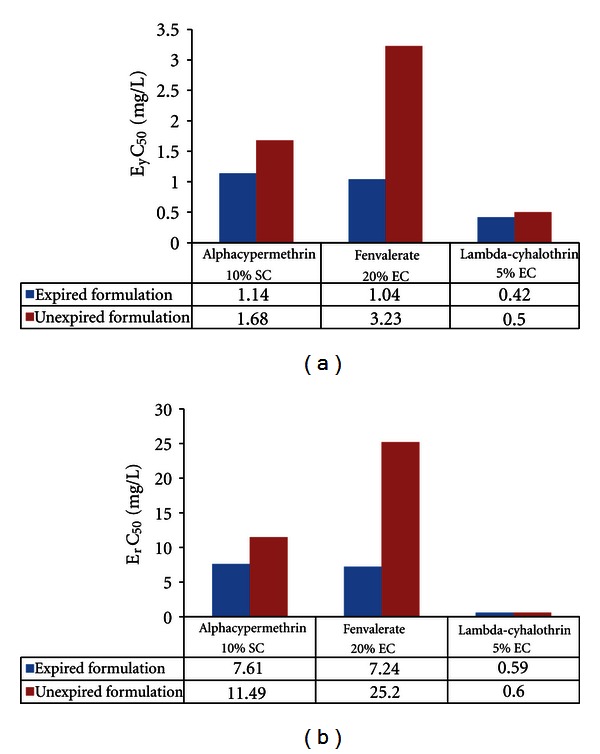
Effective yield and growth rate concentration of pyrethroids on *Pseudokirchneriella subcapitata* at 0–72 h.

**Figure 3:**
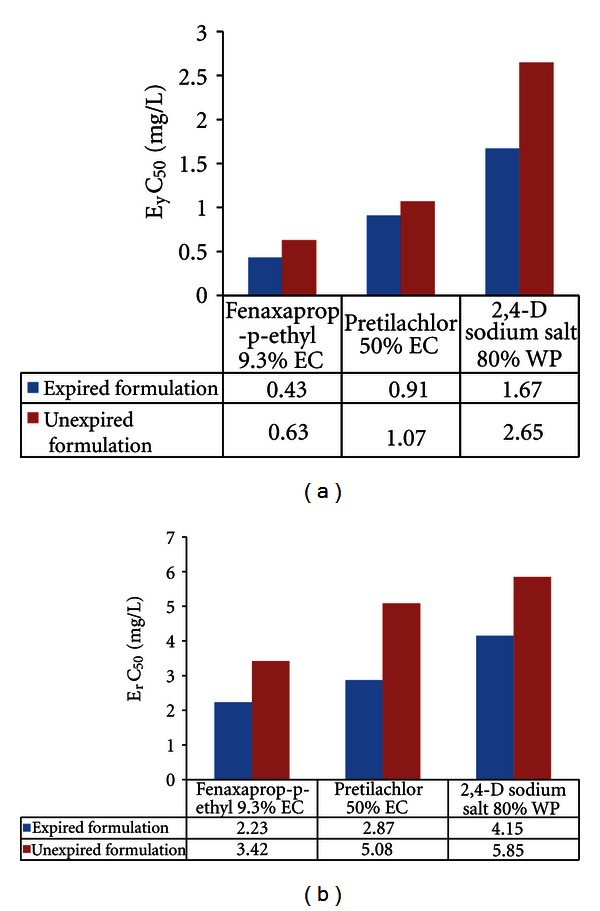
Effective yield and growth rate concentration of herbicides on *Pseudokirchneriella subcapitata* at 0–72 h.

**Figure 4:**
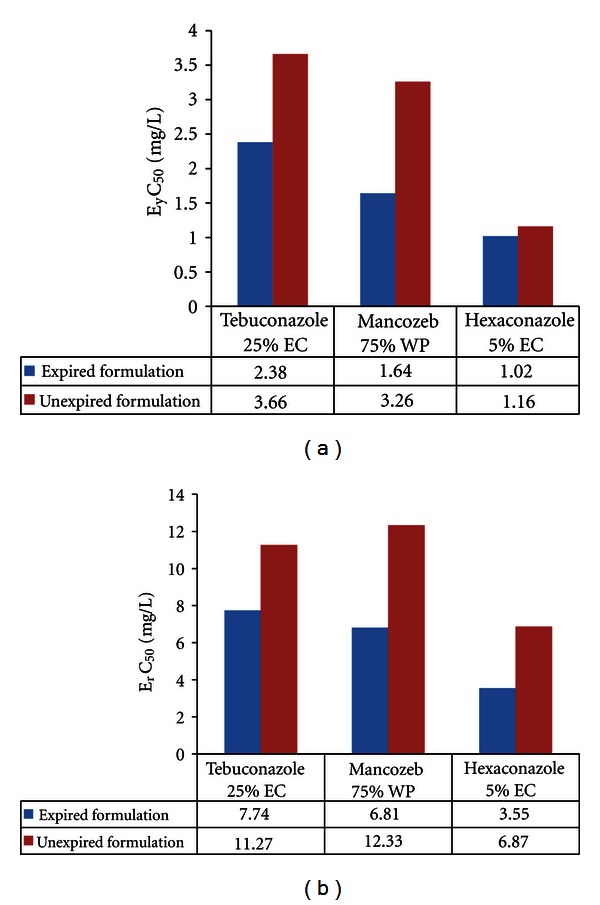
Effective yield and growth rate concentration of fungicides on *Pseudokirchneriella subcapitata* at 0–72 h.

**Figure 5:**
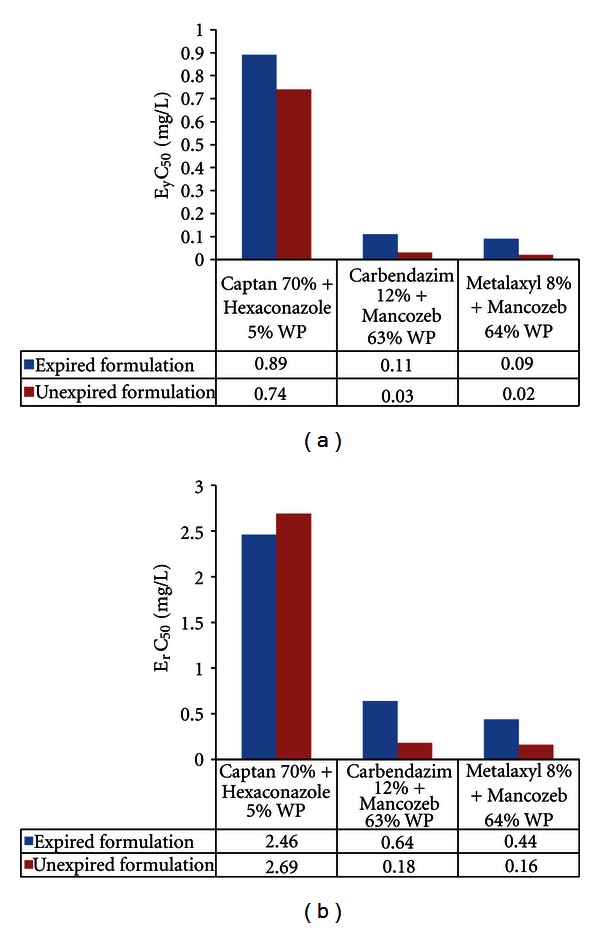
Effective yield and growth rate concentration of combinational fungicides on *Pseudokirchneriella subcapitata* at 0–72 h.

 In the present study, the biomass of *Pseudokirchneriella subcapitata *(cells/mL) in the control was increased exponentially and recorded more than 16 times in 72 h. The percent coefficient of variation (% CV) for section by section specific growth rate in the control during the whole test period did not exceed 35% and the percent coefficient of variation of average specific growth rate during the whole test period in the control did not exceed 7%. The final pH in the control did not vary by more than 1.5 units from the initial pH value.These above findings are validating the results of the present study (as per OECD guideline number 201, 2006). EC_50_ (E_y_C_50_ and E_r_C_50_) values were reported in Figures 1–5. 

 In expired condition (Table 4), insecticides and combinational fungicides were relatively less toxic when compared to the unexpired. The increased toxicity of expired pesticide formulations of pyrethroids, herbicides, and fungicides was attributed to the degraded products of active ingredients or impurities of the pesticide formulations which might be more toxic than their parent compounds, in similar low toxicity of insecticides and combinational fungicides when compared to the unexpired which may be due to less potent degraded products than their parent compounds. Along with this we have determined pH of the above pesticide formulations in expired and unexpired condition. pH of the above compounds was reported in the research article “Toxicity effect of expired pesticides to freshwater fish, *Labeo rohita*” [27]. Due to pH alteration there may be formation of unknown compounds which were not studied and there is a requirement for detailed investigation of those degraded products. The degree of toxicity depends upon the nature of the pesticides, their environmental concentration, and factors such as temperature, humidity, pH, and oxygen concentration [28]. The impurities may also contribute to the toxicity of the pesticide or might alter the physical properties of the product [29]. Ambrus et al. [29] reported that unfavorable storage conditions may lead to the decomposition of pesticides to produce degradation products much more toxic than the active ingredient. In agreement with Ambrus et al., Sanyal and Dureja [30] reported that Technical quinalphos stored in open glass bottle at 30°C for 6 months underwent degradation to give a black viscous mass containing the parent compound (C_2_H_5_O)_2_P(S)OQ (7.5%), isoquinalphos (C_2_H_5_O)_2_P(O)SQ (5.8 %), quinalphos oxon (C_2_H_5_O)_2_P(O)OQ (3.2%), 2-hydroxyquinoxalin QOH (12%), quinoxalin-2-thiol QSH (18%), diquinoxalin-2-yl-sulfide QSQ (15%), diquinoxalin-2-yldisulfide QSSQ (7%), dithienobisquinoxalin Q(S_2_)Q (2.0%), and at least 11 other compounds

 From the above study, we found that expired insecticides (include organophosphate and cyclodiene) EC_50_ values were high which indicated less toxicity when compared with unexpired and this might be due to less potent degraded products which may be formed from the active ingredients or impurities. Similar to this was reported by Hamilton et al. [31], aldicarb sulfone and aldicarb sulfoxide, metabolites of aldicarb (insecticide) whose toxicity was altered (less potent and equipotent, resp.) from parent compound. Larson et al. [32] reported that endosulfan sulfate, a transformed product of endosulfan, is more toxic to fish than endosulfan. He also reported that 3-methyl-4-aminophenol and 1-naphthol, metabolites of fenitrothion and carbaryl (insecticides-organophosphate and carbamate), respectively, are more toxic to fish than their parent compounds. Allender and Keegan [33] reported that one 2-year-old sample containing 13.8% TCP, 0.65% sulfotep, and trace amounts of chlorpyrifos oxon was reported as the cause of the death of 50 bulls treated directly with the product for ecto-parasite control [33]. In our results, expired pyrethroids EC_50_ values were lower when compared with unexpired indicating increased toxicity of expired pesticides. Lee and Jones [22] reported that pyrethroid-based pesticides have been found to cause aquatic life toxicity in storm water runoff and other runoff/discharges from urban and agricultural areas where they have been applied. These pesticides accumulate in sediments following runoff events, causing sediment toxicity. In sediment toxicity there is an accumulation of dead algae, which create anoxic conditions in sediments through their decay. This leads to an accumulation of ammonia in the sediments, which is toxic to a number of forms of aquatic life. It also leads to low DO (dissolved oxygen) conditions, which are also toxic to many forms of aquatic life [22]. 

 We found EC_50_ values of expired fungicides were lower which was due to higher toxicity against unexpired. In agreement with this, Shen [34] reported degradation product of fungicide chlordimeform, 4-chloro-methyl aniline, possesses strong carcinogenic effect towards rats than their parent substance. Herbicides in expired condition showed increased toxicity with low EC_50_ values in comparison to its counterpart. In contrast to this, Glenn [35] reported that atrazine, a herbicide, was significantly more toxic to green algae than its degradation products, deethylated atrazine, deisopropylated atrazine, hydroxy-, and diaminoatrazine. In agreement with our results, Battaglin and Fairchild [36] reported water in midwestern streams during spring and early summer runoff events can contain pesticides in sufficient quantities to be toxic to nontarget aquatic organisms and say that herbicide degradation can substantially increase the estimated toxicity of stream water to aquatic plants. Miller et al. [37, 38], through toxicity identification evaluation procedures, determined that a number of unidentified algal toxicants in Central Valley waters are likely to be herbicides. These herbicides can affect algal populations that in turn can affect DO depletion in the DWSC (deep-water ship channel) through reducing the magnitude of the algal-related oxygen demand. Based on the above data, we can understand that due to agriculture runoff pesticides able to cause algal toxicity which can also be possible in expired pesticide usage and agriculture runoff and unknown degraded products formed may further increase the toxicity level to algae and whole food chain can get disturbed and in turn leads to environment pollution.

## 5. Conclusion 

We found that E_y_C_50_ (0–72 h) and E_r_C_50_ (0–72 h) values (derived statistically) of above-mentioned pesticide formulations were higher in expired condition (pyrethroids, herbicides, and fungicides) when compared to unexpired respective formulation. When we compare this entire data as a whole, it suggests us not to use, accumulate, or store expired pesticides as bulk or individual which causes toxicity to green algae after leakage into water and leads to disturbance in the food chain and causing environment pollution. Hence, we suggest that strict regulations may be followed to ensure that expired pesticides are never released in the environment and they are properly incinerated or disposed off without impairing the aquatic environment.

## Figures and Tables

**Table 1:** Details of pesticide formulations.

Name of the pesticide	Batch number	Date of manufacture	Date of expiry
Insecticides			

Dichlorvos 76% EC (unexpired)	DH3SCR7607	April, 2010	April, 2012
Dichlorvos 76% EC (expired)	A27032	November, 2007	November, 2009
Endosulfan 35% EC (unexpired)	31915	March, 2009	March, 2011
Endosulfan 35% EC (expired)	A27057	August, 2007	August, 2009
Quinalphos 25% EC (unexpired)	SAP9K096	November, 2009	November, 2011
Quinalphos 25% EC (expired)	A27023	August, 2007	February, 2009

Pyrethroids			

Alphacypermethrin 10% SC (unexpired)	LTA-091204	November, 2009	November, 2011
Alphacypermethrin 10% SC (expired)	4	May, 2007	April, 2009
Fenvalerate 20% EC (unexpired)	1001	April, 2009	April, 2011
Fenvalerate 20% EC (expired)	111	Septemper, 2007	August, 2009
Lambda-cyhalothrin 5% EC (unexpired)	GFKBLR0502	July, 2008	July, 2010
Lambda-cyhalothrin 5% EC (expired)	GECBLJ0501	July, 2007	July, 2009

Herbicides			

Fenaxaprop-p-ethyl 9.3% EC (unexpired)	019	July, 2009	June, 2011
Fenaxaprop-p-ethyl 9.3% EC (expired)	WHPSA	August, 2007	July, 2009
Pretilachlor 50% EC (unexpired)	058	November, 2009	November, 2011
Pretilachlor 50% EC (expired)	34	August, 2007	August, 2009
2,4 D sodium salt 80% WP (unexpired)	SSP9H022	August, 2009	August, 2011
2,4 D sodium salt 80% WP (expired)	SSP7H061	August, 2007	August, 2009

Fungicides			

Tebuconazole 25% EC (unexpired)	2007041906	May, 2007	May, 2010
Tebuconazole 25% EC (expired)	2007030402	May, 2007	May, 2009
Mancozeb 75% WP (unexpired)	1MZL2036	Dec, 2008	Dec, 2010
Mancozeb 75% WP (expired)	A27102	Septemper, 2007	Septemper, 2009
Hexaconazole 5% EC (unexpired)	011	August, 2008	July, 2010
Hexaconazole 5% EC (expired)	10	November, 2006	October, 2008

Combinational fungicides			

Captan 70% + Hexaconazole 5% WP (unexpired)	AN00530	July, 2009	July, 2011
Captan 70% + Hexaconazole 5% WP (expired)	AN00166	February, 2007	February, 2009
Carbendazim 12% + Mancozeb 63% WP (unexpired)	BHJSAF7516	February, 2010	February, 2012
Carbendazim 12% + Mancozeb 63% WP (expired)	PMC-008	April, 2007	April, 2009
Metalaxyl 8% + Mancozeb 64% WP (unexpired)	SSP9I099	Septemper, 2009	Septemper, 2011
Metalaxyl 8% + Mancozeb 64% WP (expired)	TAC18	August, 2006	August, 2008

**Table 2:** Composition of the OECD TG 201 medium.

Nutrient	Final concentration (mg/L)
NaHCO_3_ (sodium hydrogen carbonate)	50.0
NH_4_Cl (ammonium chloride)	15.0
MgCl_2_·6H_2_O (magnesium chloride)	12.0
CaCl_2_·2H_2_O (calcium chloride)	18.0
MgSO_4_·7H_2_O (magnesium sulphate)	15.0
KH_2_PO_4_ (potassium dihydrogen phosphate)	1.60
FeCl_3_·6H_2_O (ferric chloride)	0.064
Na_2_EDTA·2H_2_O (EDTA disodium salt)	0.100
H_3_BO_3_ (boric acid)	0.185
MnCl_2_·4H_2_O (manganese(II) chloride)	0.415
ZnCl_2 _(zinc chloride)	0.00300
CoCl_2_·6H_2_O (cobaltous chloride)	0.00150
Na_2_MoO_4_·2H_2_O (sodium molybdate)	0.00700
CuCl_2_·2H_2_O (copper(II) chloride)	0.00001

**Table 3:** Percent inhibition of yield (%I_*y*_) and growth rate (%I_*r*_) at 72 hours.

S. no.	Name of the pesticide	Conc. exposed (mg/L)	Percent inhibition		Percent inhibition	
			RowSpanEmpty	RowSpanEmpty	unexpired		expired	
			RowSpanEmpty	RowSpanEmpty	(%I_*y*_)	(%I_*r*_)	(%I_*y*_)	(%I_*r*_)
Insecticides						

1	Dichlorvos 76% EC	0.95	1.57	0.30	−1.05	−0.20
		3.05	11.52	2.31	3.66	0.71
		9.77	32.46	7.42	21.99	4.70
		31.25	45.03	11.30	30.37	6.84
		100.00	99.48	86.82	91.10	45.02
2	Endosulfan 35% EC	0.10	2.09	0.40	1.68	0.32
		0.31	21.99	4.70	11.17	2.27
		0.98	57.59	16.18	33.52	7.81
		3.13	84.82	35.31	67.04	21.16
		10.00	100.00	100.00	92.18	47.85
3	Quinalphos 25% EC	0.10	8.38	1.68	2.23	0.43
		0.31	16.76	3.51	12.29	2.51
		0.98	41.34	10.20	34.64	8.13
		3.13	73.74	25.45	69.83	22.83
		10.00	97.21	65.50	94.97	55.66

Pyrethroids						

4	Alphacypermethrin 10% SC	0.1	1.68	0.32	2.79	0.54
		0.31	11.17	2.27	19.55	4.16
		1.0	33.52	7.81	44.69	11.32
		3.1	67.04	21.16	75.42	26.70
		10.0	92.18	47.85	95.33	57.69
5	Fenvalerate 20% EC	0.1	0.56	0.11	5.59	1.10
		0.31	4.47	0.88	19.55	4.16
		1.0	22.35	4.84	47.49	12.31
		3.1	44.69	11.32	77.09	28.02
		10.0	81.01	31.54	96.09	59.96
6	Lambda-cyhalothrin 5% EC	0.1	1.55	0.29	4.12	0.79
		0.31	11.34	2.27	15.46	3.17
		1.0	100.00	100.00	100.00	100.00
		3.1	100.00	100.00	100.00	100.00
		10.0	100.52	100.00	100.52	100.00

Herbicides						

7	Fenaxaprop-p-ethyl 9.3% EC	0.10	1.83	0.34	8.22	1.58
		0.31	25.11	5.33	36.53	8.38
		0.98	73.06	24.09	82.19	31.61
		3.13	89.04	40.32	94.52	52.44
		10.00	99.09	79.63	100.00	100.00
8	Pretilachlor 50% EC	0.10	−1.05	−0.20	6.28	1.23
		0.31	14.14	2.88	21.99	4.70
		0.98	46.60	11.85	50.79	13.38
		3.13	84.82	35.31	92.67	48.49
		10.00	97.91	69.39	100.00	100.00
9	2,4 D sodium salt 80% WP	0.10	1.06	0.20	5.29	1.03
		0.31	7.94	1.57	16.93	3.51
		0.98	21.16	4.51	35.98	8.44
		3.13	47.62	12.23	62.43	18.49
		10.00	99.47	86.79	100.53	100.00

Fungicides						

10	Tebuconazole 25% EC	0.10	−2.23	−0.42	1.12	0.22
		0.31	2.79	0.54	9.38	1.68
		0.98	11.17	2.27	22.35	4.84
		3.13	46.93	12.11	61.45	18.19
		10.00	91.06	45.44	96.65	62.53
11	Mancozeb 75% WP	0.10	2.58	0.49	6.70	1.31
		0.31	10.31	2.05	22.16	4.72
		0.98	13.40	2.71	38.66	9.21
		3.13	46.39	11.74	58.25	16.43
		10.00	90.21	43.19	97.94	69.48
12	Hexaconazole 5% EC	0.10	5.29	1.03	13.23	2.69
		0.31	15.34	3.16	18.52	3.88
		0.98	51.85	13.82	58.20	16.49
		3.13	66.14	20.44	73.02	24.70
		10.00	97.35	65.85	100.00	100.00

Combinational fungicides						

13	Captan 70% + Hexaconazole 5% WP	0.03	1.02	0.19	0.51	0.10
		0.10	11.68	2.34	9.14	1.80
		0.31	25.38	5.50	22.34	4.75
		0.98	62.44	18.36	44.67	11.11
		3.13	92.89	48.79	96.95	63.20
		10.00	100.51	100.00	100.51	100.00
14	Carbendazim 12% + Mancozeb 63% WP	0.003	1.02	0.19	−2.03	−0.38
		0.010	24.37	5.25	1.52	0.29
		0.03	62.44	18.36	24.37	5.25
		0.10	80.20	30.24	54.82	14.91
		0.31	96.45	60.68	62.44	18.36
		1.00	100.51	100.00	97.97	69.57
15	Metalaxyl 8% + Mancozeb 64% WP	0.002	1.15	0.22	−4.02	−0.76
		0.005	25.86	5.76	1.72	0.33
		0.02	60.34	17.74	20.11	4.32
		0.05	71.84	24.26	54.60	15.16
		0.16	86.21	37.68	86.21	37.68
		0.50	99.43	86.58	98.28	73.16

Same concentrations were used for both unexpired and expired pesticide formulations exception for Metalaxyl 8% + Mancozeb 64% WP (expired). Concentrations of Metalaxyl 8% + Mancozeb 64% WP (expired) are 0.003, 0.010, 0.03, 0.10, 0.31, and 1.00 mg/L.

**Table 4:** Toxicity level of expired pesticide formulations against unexpired in percentage (%).

	Toxicity level (%)
Insecticides	
Dichlorvos 76% EC	—
Endosulfan 35% EC	—
Quinalphos 25% EC	—
Pyrethroids	
Alphacypermethrin 10% SC	32.14
Fenvalerate 20% EC	67.80
Lambda-cyhalothrin 5% EC	16.00
Herbicides	
Fenaxaprop-p-ethyl 9.3% EC	31.75
Pretilachlor 50% EC	14.95
2,4-D sodium salt 80% WP	36.98
Fungicides	
Tebuconazole 25% EC	Nearly two fold higher than unexpired
Mancozeb 75% WP	Nearly two fold higher than unexpired
Hexaconazole 5% EC	Nearly two fold higher than unexpired
Combinational fungicides	
Captan 70% + Hexaconazole 5% WP	—
Carbendazim 12% + Mancozeb 63% WP	—
Metalaxyl 8% + Mancozeb 64% WP	—
